# Supplementary material for: The complete structure of an activated open sodium channel
Source: Nat Commun. 2017 Feb 16;8:14205. doi: 10.1038/ncomms14205 (PMC5316852; doi:10.1038/ncomms14205)
Supplement: Supplementary Information — Supplementary Figures, Supplementary Tables. [file ncomms14205-s1.pdf]

Channel	Act. $V_{1/2}$ (mV)	Inact. $V_{1/2}$ (mV)	Z (e^-)	Rec. inact. τ (ms)
**NavMs WT**	-87 ± 3	-127 ± 3	-3.4 ± 0.2	223 ± 18
**W77Y**	-75 ± 2*	-112 ± 4*	-2.3 ± 0.3*	217 ± 17
**W77F**	-71 ± 3*	-105 ± 4*	-2.2 ± 0.3*	225 ± 14

Supplementary Table 1: Biophysical properties of the NavMs channels (based on data in Figure 5)
Values of voltage dependence of activation (Act. $V_{1/2}$), voltage dependent inactivation (Inact. $V_{1/2}$), Z, estimated charge (Z e-) as measured by the slope of the conductance voltage relationship, and rate of recovery from inactivation (Rec. Inact. τ) for wildtype NavMs and the W77Y and W77F mutants. Asterisk indicates $P<0.05$ based on a two tailed Student's T-test.

Mutation	Forward 5'-3
**I218C**	ATTGGCATTTGTGTAGATGCAATGGCAATCACCAAG
**W77F**	CCGCAGCGGCTTCAATCTGTTTGATTTTGTG
**W77Y**	CCGCAGCGGCTACAATCTGTTTGATTTTGTG
**W77A**	CCGCAGCGGCATGAATCTGTTTGATTTTGTG
**W77M**	CCGCAGCGGCGTCAATCTGTTTGATTTTGTG

Supplementary Table 2: Mutagenic primers [synthesized in duplex] used to create the electrophysiological constructs.

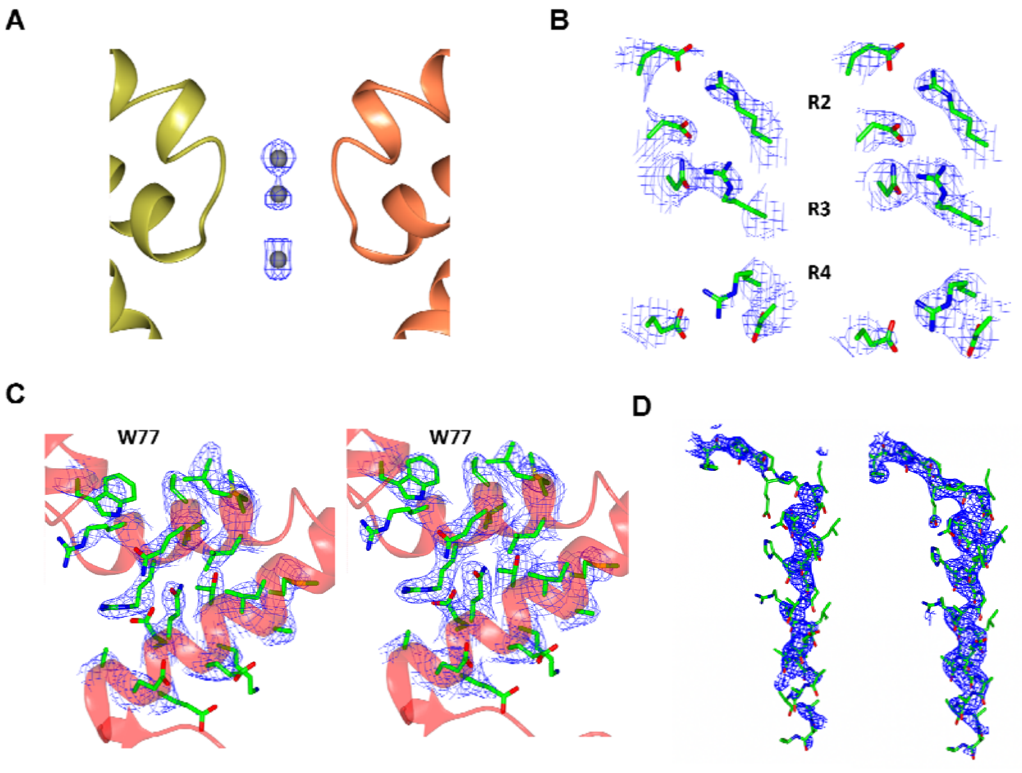

<u>Supplementary Fig 1:</u> Electron density maps of the 2.45 A resolution wildtype NavMs structure

A) Annealed omit map (1.3 sigma) of the three sodium ions (grey spheres) showing the quality of the refined structure. Panels B-D include pairs of annealed omit maps (left, 1.1 sigma) and 2Fo-Fc maps (right: 0.8 sigma). B) The region around the VS arginines (R2-R4); C) The region around the interaction domain, including the linker, bottom of S6, and residue W77 in S3; D) the CTD helix.

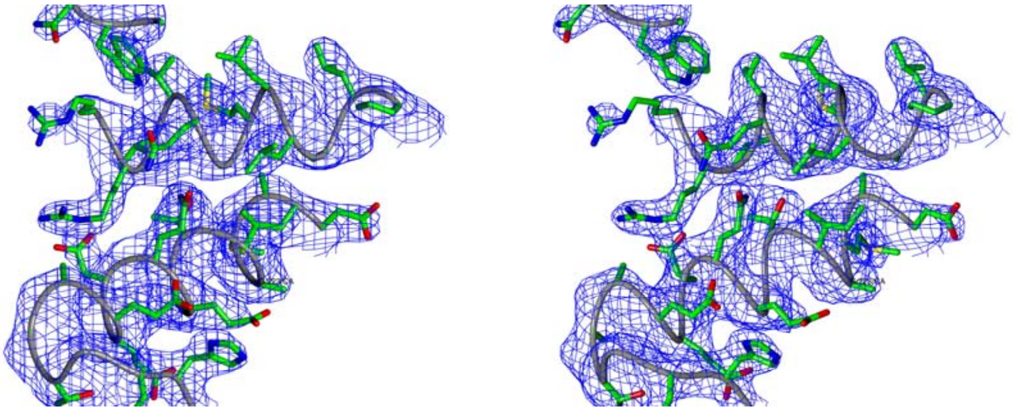

<u>Supplementary Fig 2:</u> Stereo view of the electron density map of the 2.45 A resolution wildtype NavMs structure showing the quality of the map.

This figure shows the quality of the 2Fo-Fc map (contoured at 1.3 sigma) around the important interaction domain (including the S3 tryptophan, L4-L5 linker and near the end of the S6 helix) [comparable to the central panel in Fig 4a].

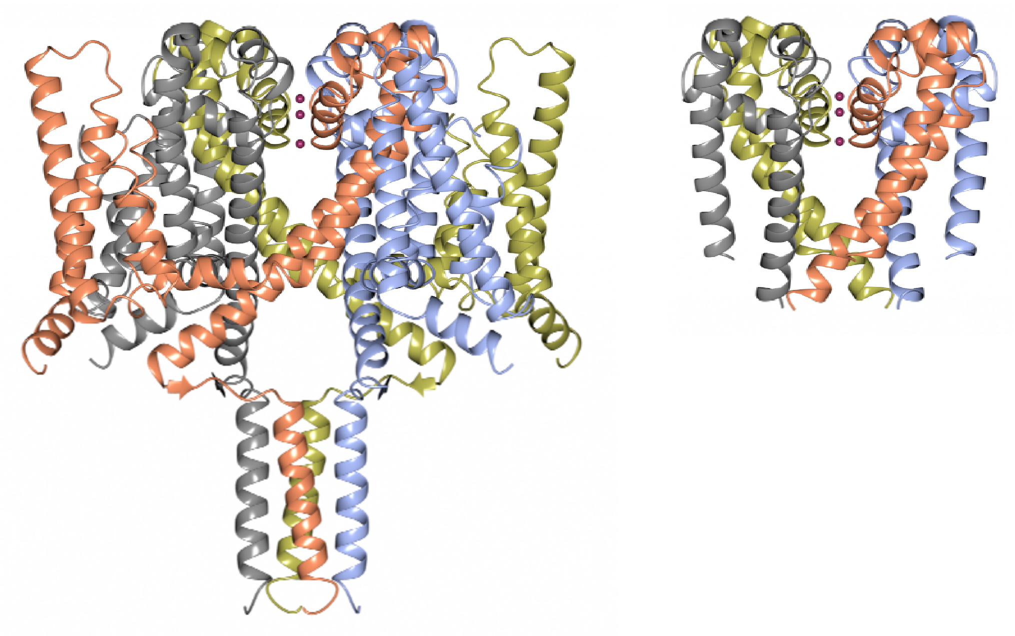

<u>Supplementary Fig 3:</u> Structure comparisons of the full-length and pore-only constructs of the NavMs channel

Comparison of the NavMs channel (left) and pore-only (right) constructs, with the four chains in different colours, drawn in ribbon motif, and the three sodium ions as purple balls.

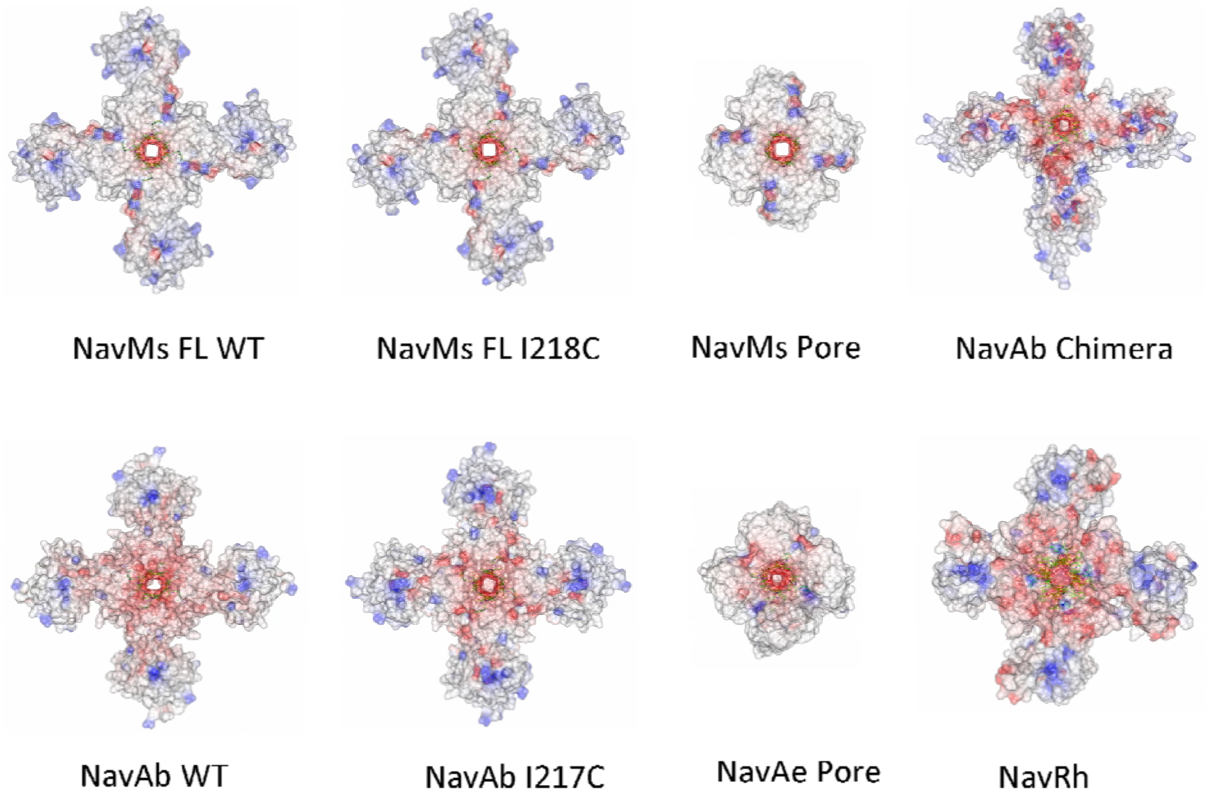

<u>Supplementary Fig 4</u>: Comparisons of the pore gates in sodium channel structures
Comparison of space filling models viewed from the extracellular surface, showing the relative size of the pore hole and the electrostatic surface for NavMs channel (wild type and I218C mutant), NavMs pore-only construct, NavAb chimera, NavAb (wild type and I217C mutant), and NavAe1 pore-only construct, and NavRh.

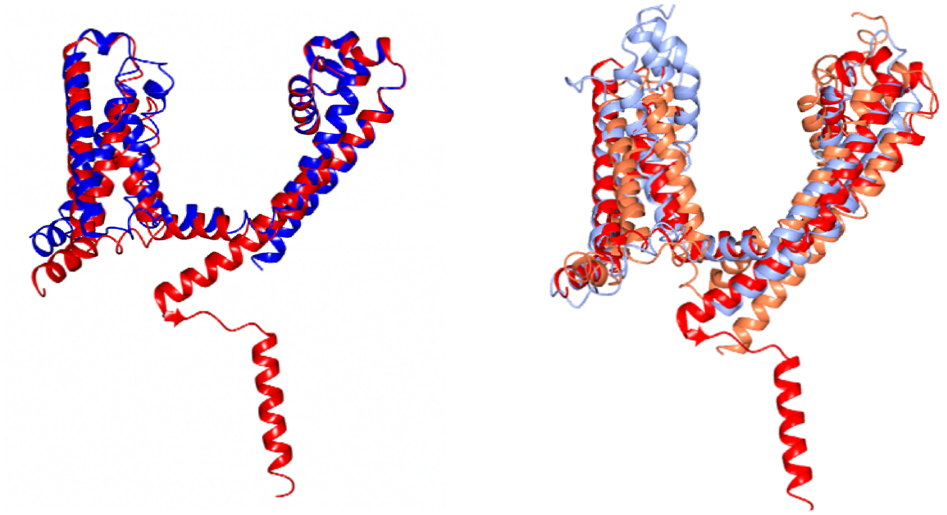

<u>Supplementary Fig 5:</u> **Alternate overlays of NavMs (open) and NavAb (closed) structures, and overlays of NavMs with other ion channel structures**
Left: Overlay of the SF regions (compare with the overlay of VS regions in Figure 4) as an alternate way of comparing the NavMs open activated structure (red) and the NavAb closed pre-activated structure (blue). Regardless of which way the structures are overlaid, the resulting linker displacement produces a further change in the S5 and S6 helices, producing a wider pore gate in NavMs.
Right: Overlay of the SF regions of NavMs (red) and other ion channels, TPC1 (orange) and the Kv1.2-Kv2.1 paddle chimera (blue) structures showing that the resulting interactions and dispositions of the voltage sensor domains relative to the pore domains vary across different channels, as do the positions of the S4-S5 linkers relative to the pore domain, and their resulting impact on opening the S6 pore gate.

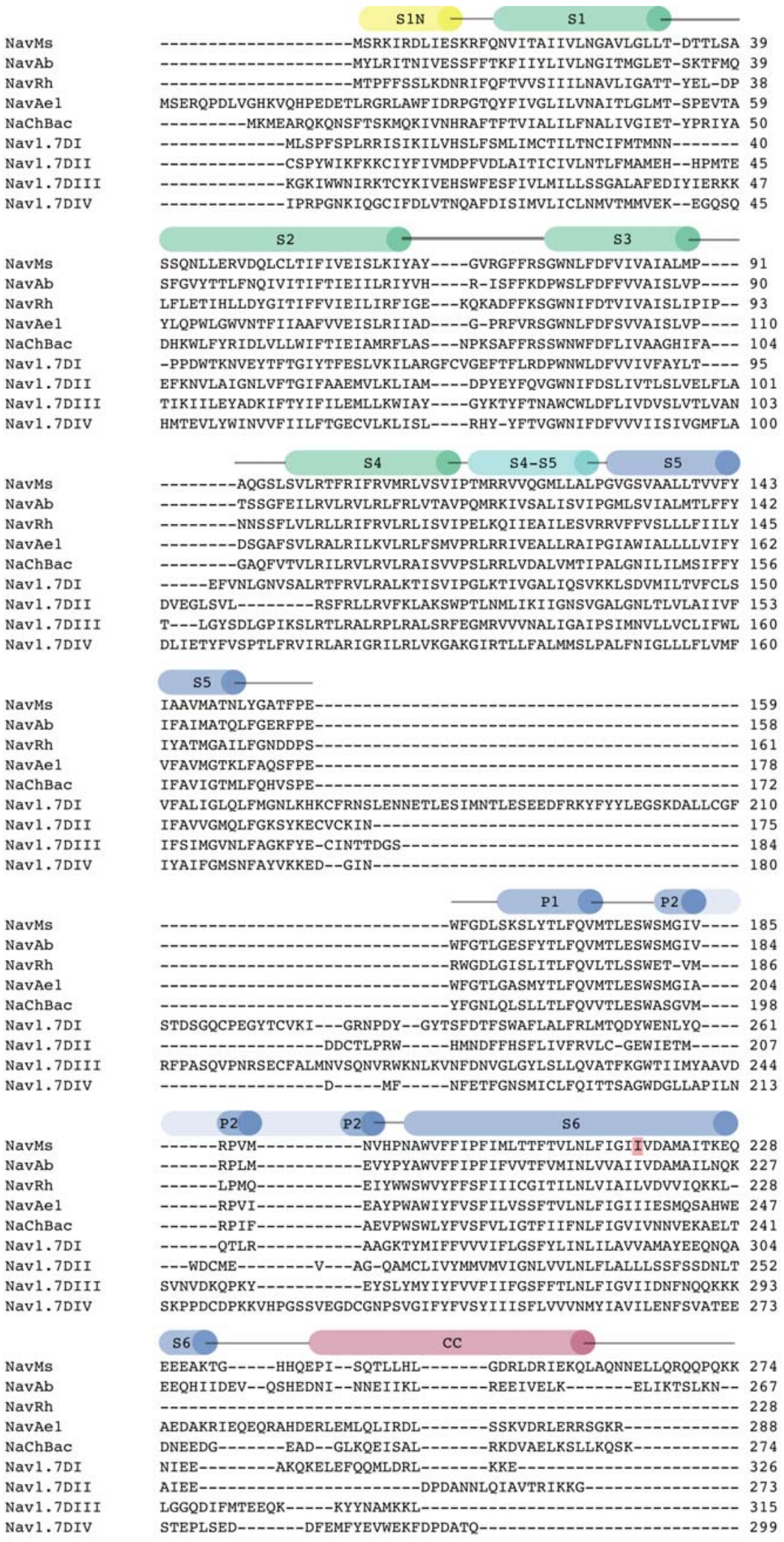

Supplementary Fig 6: Sequences of sodium channels
Complete sequence alignments of NavMs, NavAb (the location of the I218C mutation is indicated by the red box), NavRh, NavAe1, NaChBac, and human Nav1.7 (all domains).

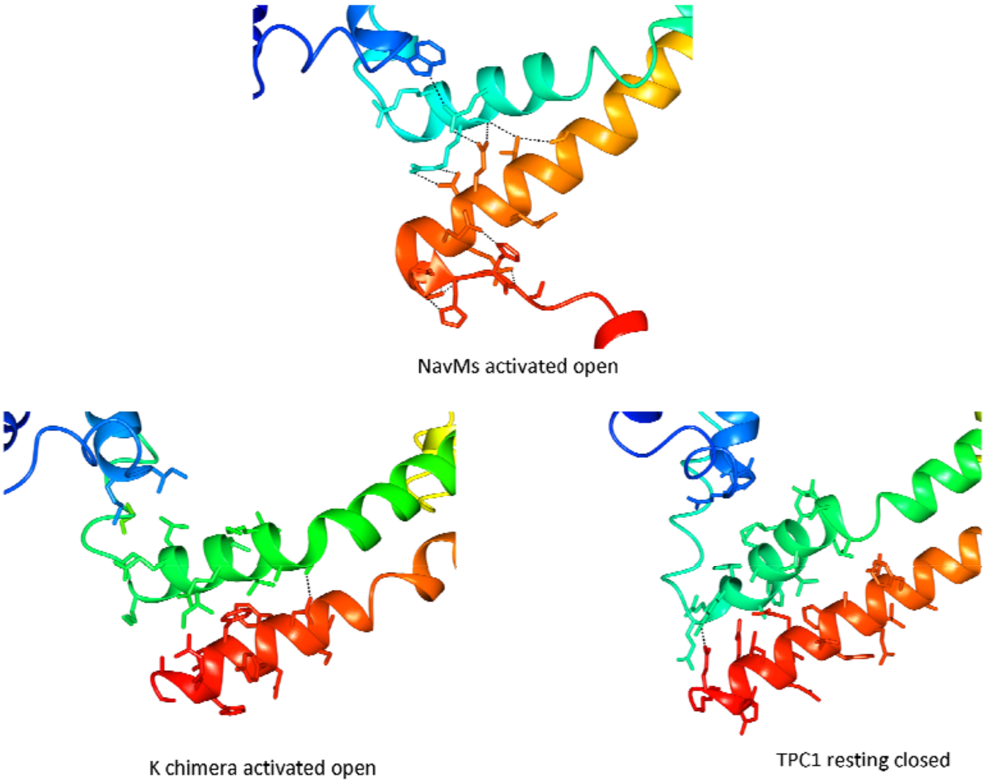

<u>Supplementary Fig 7:</u> Comparison of the NavMs interaction motif with equivalent regions of other ion channels

Zoomed views of the NavMs interaction motif, with the equivalent regions of the K chimera (open) and TPC1 (closed) structures, indicating how different the VS/pore gate interactions are in the different ion channels. Whilst at the end of the sodium channel S6 transmembrane domain there is an extensively H-bonded and salt-bridged interaction domain [6 such interactions –highlighted in the black circles] involving the VS S3 helix (and its conserved W77), as well as the S4-S5 linker, the TPC1 and K chimera structures have only minimal interactions involving the S6 gate region and the S4-S5 linker [in each case, only a single stabilising H-bond], and are missing any interaction with S3 (and, importantly lack the corresponding W in their sequences). The Trp residue is completely conserved within the sodium channel family but not in any of the other ion channels. The angle of the S6 helix relative to the S4-S5 linker producing the lever action in NavMs (which enables opening) is also very different from the other structures. Furthermore, the EEE sequence in NavMs that was previously shown to be functionally-important in NavMs[6], is involved in the extensively H-bonded cluster. The K chimera does have an EE in the C-terminal region of its sequence but it is in a structurally unrelated area, lying beyond the end of the visible part of S6, as well as beyond the region in NavMs that forms the interaction motif. TPC1 also has an E-rich sequence in its CTD, but it forms a single H bond which is not equivalent to any of the ion pairing/H-bonds in the open NavMs structure. Thus the interaction domain of the sodium channel appears to be unique and associated with the channel opening mechanism.

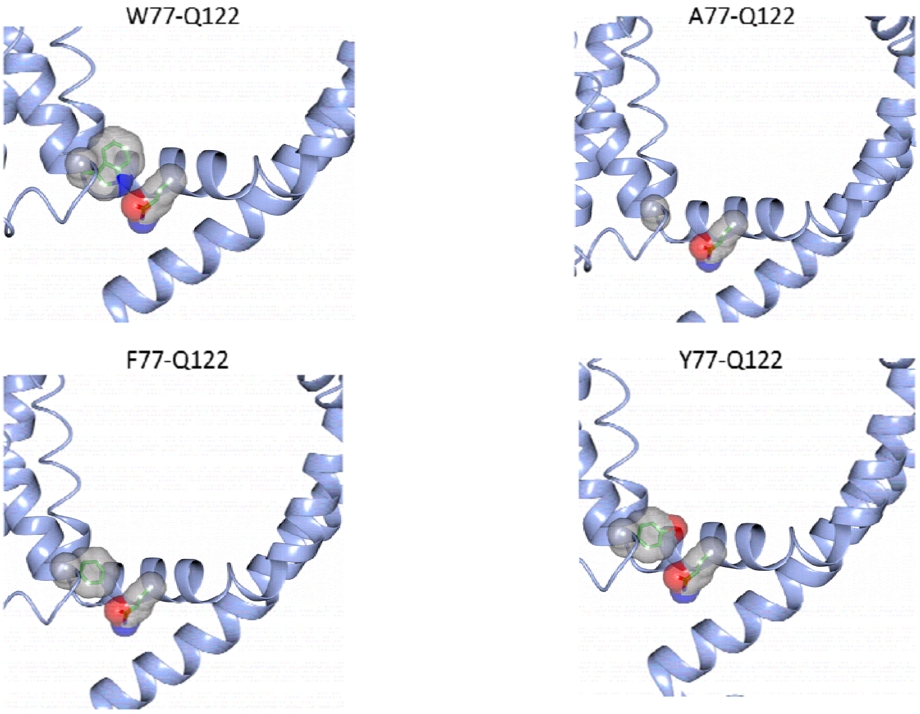

<u>Supplementary Fig 8:</u> Homology models of the effects of W77 mutations on the interaction motif

Left, top: The crystal structure of the wild type NavMs (W77), highlighting the interaction motif region (depicted as ribbons) with the side chains of the W77 and Q122 residues shown in space filling/CPK colours.

Right, top and Left and Right, bottom: Homology models of the W77A, W77F and W77Y mutants, showing that different interactions of these with the Q122 side chains could result in less stabilised open structures.
